# Retraction: *Flaviviridae* RdRp exploits NSUN2-driven m^5^C methylation to establish persistent infection

**DOI:** 10.1371/journal.ppat.1014764

**Published:** 2026-11-09

**Authors:** The PLOS Pathogens Editors

After this article [1] was published, concerns were raised about results presented in Figs 1, 3, S6, S8, S10, and S21.

Specifically:

- Fig 1E Lung β-actin panel appears similar to Fig 3G WCL β-actin panel.
- There appear to be similarities between parts of the following the following confocal microscopy line graphs:
  ○ Fig S6F NS4B and Fig S6F NS5A.
  ○ Fig S8A NS1 and Fig S8A NS2B.
- Within the following confocal microscopy line graphs, there appear to be repetitive areas:
  ○ Fig S8B NS3.
  ○ Fig S8B NS4B.
  ○ Fig S8B NS5A.
  ○ Fig S8C Vector.
  ○ Fig S10B CSFV-NS5B.
  ○ Fig S10E ML-792.
- The following confocal microscopy line graphs in Fig S8A appear to show multiple y values for the same x value:
  ○ NS3.
  ○ NS4A.
  ○ NS4B.
- Fig S21B Npro panel in [1] appears similar to Fig 8B Npro panel in [2] despite representing different experimental conditions.

The corresponding author stated that the Fig 1E Lung β-actin panel is incorrect and provided underlying data and a replacement panel.

Regarding the concerns for the confocal microscopy graphs, the corresponding author provided replacement graphs plotted from reanalysis of the original confocal microscopy images. Upon editorial review, the data provided did not resolve these concerns.

The corresponding author stated that the Fig S21B panel in [1] is incorrect, and provided a replacement panel and underlying uncropped blot data. The underlying blot provided raised additional concerns with the results in Fig S21B.

In light of the above concerns that question the reliability and integrity of the reported results, the *PLOS Pathogens* Editors retract this article.

All authors did not agree with the retraction and stand by the article’s findings.

In addition to the above issues, concerns were raised about discontinuities in the background in multiple western blot panels in [1]. Due to the above unresolved concerns, PLOS did not investigate the blot discontinuity concerns further.
